# Diffusion MRI of the Body: Beyond PGSE‐EPI

**DOI:** 10.1002/jmri.70451

**Published:** 2026-07-14

**Authors:** Matthew T. Cherukara, Wenbo Sun, David Leitão, Shaihan Malik, Radhouene Neji, Joseph V. Hajnal, Andrada Ianus

**Affiliations:** ^1^ School of Biomedical Engineering and Imaging Sciences King's College London London UK

**Keywords:** body imaging, data acquisition, diffusion imaging, distortion correction, fast acquisition

## Abstract

Diffusion magnetic resonance imaging (dMRI) is a widely used clinical imaging technique which is sensitive to changes in tissue microstructure including cellularity, perfusion, and tissue damage. DMRI has been used in whole‐body imaging as well as in targeted imaging of every major anatomical region. Most clinical dMRI applications use a standard acquisition technique: the pulsed gradient spin echo (PGSE) preparation with echo planar imaging (EPI) readout. PGSE creates strong diffusion contrast while removing the effect of *T*_2_*, and EPI enables imaging of an entire 2D slice in a single shot. The PGSE‐EPI method is rapid and robust; however, it is limited by low resolution and distortions typically caused by motion and susceptibility artifacts. Since the inception of dMRI, other methods have been used to sensitize diffusion and to read out the signal, which overcome different weaknesses of PGSE‐EPI. These methods have largely been developed and used in the brain, but many of them have been applied to body dMRI as well. This review describes the major families of non‐standard techniques used in body dMRI, covering both diffusion encoding strategies as well as image readout strategies. It then explores how these techniques have been applied in non‐brain clinical applications and assesses the strengths and benefits of each method in various clinical contexts. Non‐standard acquisition techniques have the potential to improve the value and efficiency of dMRI in the body, and further work to standardize and validate these sequences will enable their use in clinical contexts.

**Evidence Level:** 3.

**Technical Efficacy:** 2.

## 1 Introduction

Diffusion magnetic resonance imaging (dMRI) is a family of MRI techniques that exploit signal changes arising from the random movement of water molecules to characterize tissue microstructure [1]. Today, dMRI is widely available and is a recommended component of clinical imaging protocols in a wide range of anatomies and pathologies [2, 3, 4, 5, 6]. The process of sensitizing the MR signal to water diffusion was first demonstrated by Stejskal and Tanner in 1965 [7], with the pulsed‐gradient spin‐echo (PGSE) sequence. Feasible acquisition times and robustness against bulk motion were achieved with the use of echo planar imaging (EPI) readout in 1990 [8]. Since then, PGSE‐EPI has become ubiquitous for clinical dMRI throughout the body [9, 10, 11]. It is not, however, without limitations, mostly related to its remaining sensitivity to motion, its susceptibility to distortion, and its lack of specificity to complex underlying tissue microstructures [12, 13].

To overcome these and other limitations of PGSE‐EPI, advanced techniques have proliferated for every step in the dMRI acquisition pipeline. Recent Reviews of dMRI techniques have focused on a single anatomical region and/or clinical presentation, and tend to span data acquisition, processing, analysis, and clinical findings of dMRI in the chosen application [3, 4, 5, 6, 14, 15, 16].

This article reviews the use of non‐PGSE‐EPI acquisitions of dMRI throughout the body. It aims to describe the techniques currently in use (Section 3), and review how they are applied different anatomical regions are reviewed (Section 4). The findings are synthesized in Section 5. This article's scope is limited to studies that image adult humans, using the non‐standard techniques described below.

## 2 Diffusion MRI With PGSE‐EPI

### 2.1 Diffusion Encoding With PGSE

**FIGURE 1:**
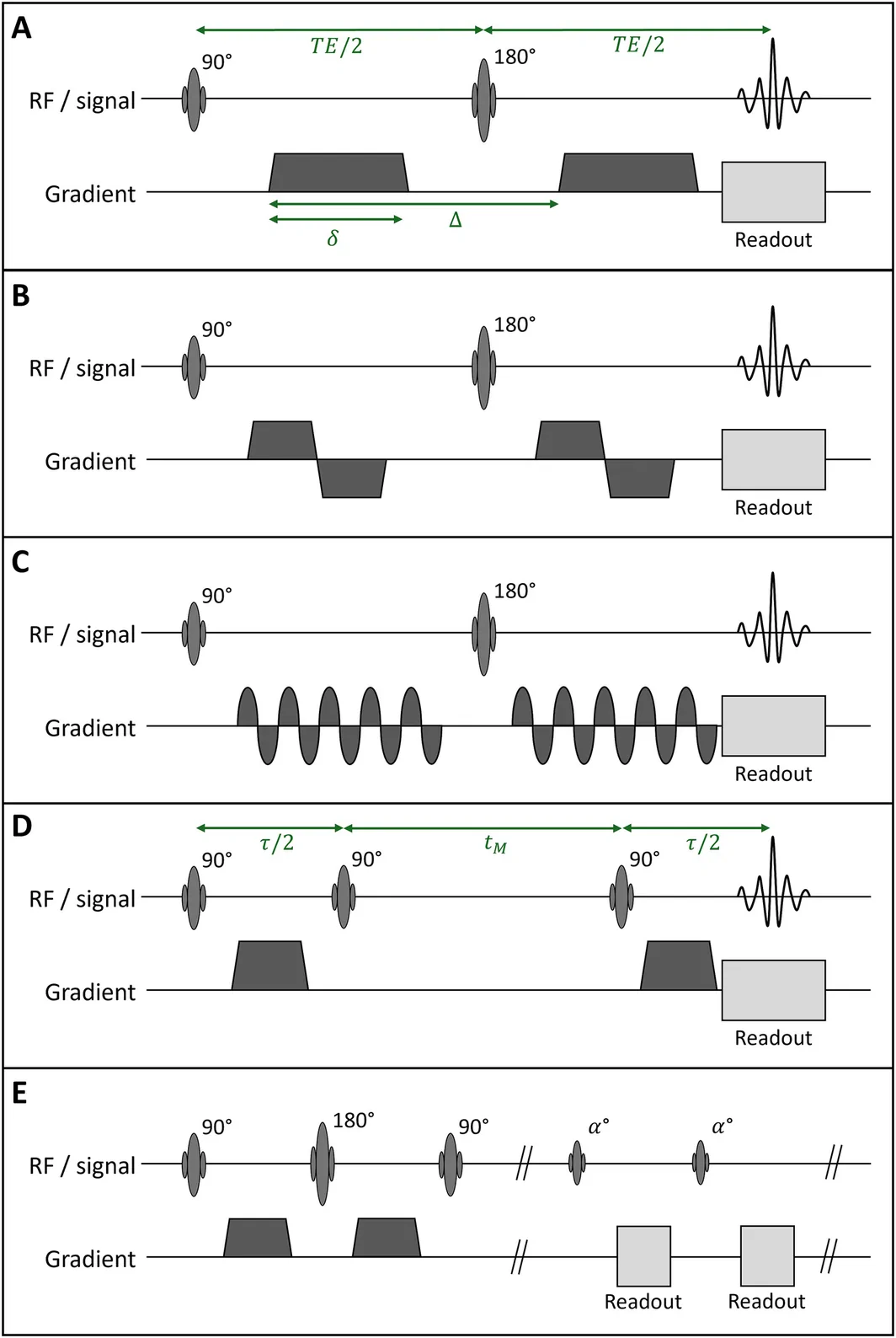
Simplified pulse sequence diagrams for diffusion sensitization and refocusing schemes. For simplicity, diffusion gradients are shown in only one direction, and image encoding gradients are not shown. (A) Pulsed‐gradient spin echo (PGSE). (B) Bipolar gradient spin echo. (C) Oscillating gradient spin echo. (D) Stimulated echo (STE). (E) Driven equilibrium steady‐state free precession (SSFP).

The standard sequence for dMRI is Stejskal and Tanner's PGSE sequence [7], which is schematically represented in Figure 1A. It begins with a spin‐echo (SE) sequence, with a symmetrical pair of field gradient pulses added on either side of the 180° refocusing pulse [7]. In the case of static spins, the dephasing effect of the first gradient pulse is exactly reversed by the second; but if a spin has moved (with respect to the direction of the pulsed gradients) in the interval between the pulses, Δ, it will accrue a net phase and contribute to signal loss. For Gaussian diffusion, the signal loss can be described by:

(1) $$S=S_{0}\cdot e^{-b\cdot D}$$

where $S_{0}$ is the signal that would be measured in the absence of diffusion sensitization, b is the diffusion weighting, which depends on the specifics of the diffusion‐sensitizing gradients, and D is the diffusion coefficient, which depends on the thermal properties of the sample and its microscopic structure [1]. For idealized rectangular gradients of magnitude G, duration δ, and separation Δ, we obtain [7]:

(2) $$\begin{array}{c} b={\left(\mathrm{\gamma G\delta}\right)}^{2}\left(\Delta -\frac{\delta }{3}\right) \end{array}$$

where γ is the proton gyromagnetic ratio. Gradient slew rates mean that rectangular gradients are not achievable in practice, so a modification for trapezoidal gradients is used for *b*‐value calculations in practice.

Due to microstructural barriers, diffusion in biological tissues deviates from free Gaussian displacement. Nevertheless, in the limit of moderate diffusion weighting, b, the signal decay is still effectively captured by a monoexponential function (Equation 1), albeit with D replaced by apparent diffusion coefficient (ADC), which aggregates voxel‐level restriction and hindrance, and serves as an experimental observation dependent on sequence timing, rather than as an intrinsic physical constant.

While widely used, the PGSE preparation is not without its disadvantages. For example, strong diffusion encoding (high *b*‐value) requires long diffusion time Δ. This means that it is highly sensitive to bulk flow or motion of the tissue, and limits its applicability in anatomical regions with high motion, such as the heart [12]. Furthermore, long Δ (and hence, long TE) means that the measured signal is also subject to *T*_2_ decay, which is especially a problem in tissues such as the myocardium [17].

### 2.2 Standard Diffusion Models

In tissues where cells are laid in linear structures, such as the myocardium or muscle tissue, diffusion will be significantly anisotropic [17]. Here, Equation (1) describing Gaussian diffusion is further modified and the scalar ADC is replaced with a three‐dimensional diffusion tensor:

(3) $$\begin{array}{c} \bar{D}=\left[\begin{array}{ccc} D_{\mathrm{xx}} & D_{\mathrm{xy}} & D_{\mathrm{xz}}\\ D_{\mathrm{yx}} & D_{\mathrm{yy}} & D_{\mathrm{yz}}\\ D_{\mathrm{zx}} & D_{\mathrm{zy}} & D_{\mathrm{zz}} \end{array}\right] \end{array}$$

From this, one can calculate: mean diffusivity (MD—the trace of $\bar{D}$), which approximates an isotropic diffusion coefficient; fractional anisotropy (FA—the variance of the eigenvalues of $\bar{D}$), which indicates the directionality of diffusion in the tissue; and other directional parameters. Fully characterizing $\bar{D}$ using diffusion tensor imaging (DTI) requires acquiring at least six diffusion‐weighted images, with G applied in different directions, in addition to a b=0 image [18].

Further advancements to dMRI modeling have been proposed, aiming to increase the specificity of dMRI metrics to the underlying tissue microstructure, and have been recently reviewed elsewhere [19]. These include intravoxel incoherent motion (IVIM), which modifies Equation (1) into a biexponential model with two pools; and diffusion kurtosis imaging (DKI), which adds an excess kurtosis parameter to the mono‐exponential model. Given that such techniques mostly employ standard acquisitions, they are beyond the scope of this review.

### 2.3 Image Acquisition With Single‐ and Multi‐Shot EPI

**FIGURE 2:**
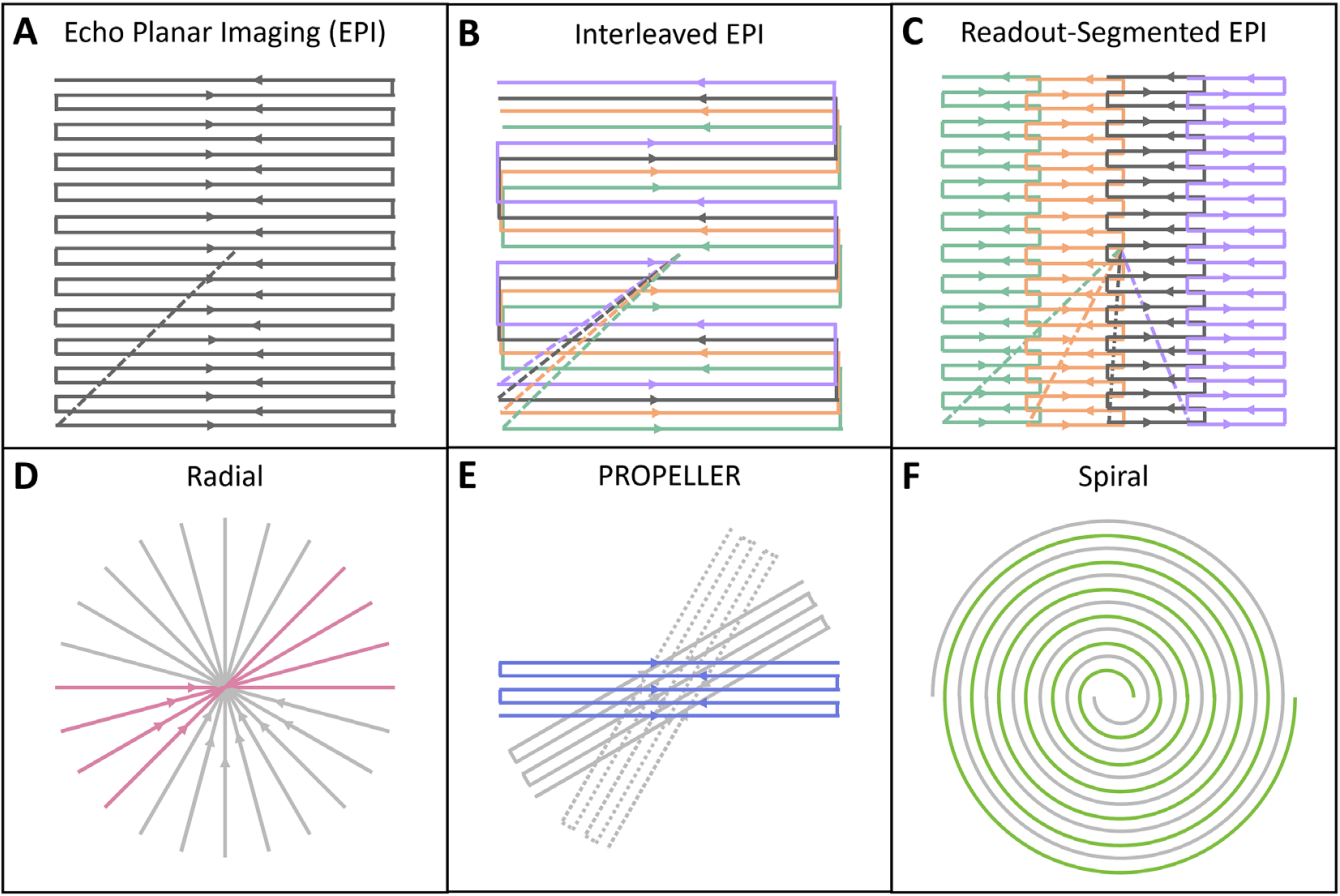
Simplified k‐space trajectories for 2D image readout methods. (A) Echo‐planar imaging (EPI). (B) Interleaved EPI (also known as multi‐shot EPI) with four leaves. (C) Readout‐segmented EPI, with four segments. (D) Radial. (E) Periodically rotated overlapping parallel lines with enhanced reconstruction (PROPELLER). (F) Spiral, with two interleaves.

The final element of the dMRI sequence is image acquisition, and echo planar imaging (EPI—Figure 2A) is by far the most utilized technique [8, 20]. EPI enables acquisition of the whole slice in a single shot, so bulk motion at a timescale close to TR is not an issue. The short TR also means that dMRI volumes can be acquired in reasonable time, including advanced diffusion techniques.

On the downside, the long readout duration of EPI makes it vulnerable to $T_{2}^{*}$‐blurring and geometric distortions, as well as limiting the achievable resolution. These can be mitigated by traversing the phase‐encoding direction of *k*‐space ($k_{\mathrm{PE}}$) more quickly. One method for this is interleaved EPI (Figure 2B), in which regularly spaced lines of k‐space are acquired in subsequent shots [21]. This means that the whole of $k_{\mathrm{PE}}$ is traversed more quickly, albeit at the expense of lower effective *k*‐space resolution within a given shot. An alternative method is to partition *k*‐space into segments in the readout direction ($k_{\mathrm{RO}}$) and acquire one segment per shot, with full‐density sampling in $k_{\mathrm{PE}}$, known as readout‐segmented EPI (RS‐EPI—Figure 2C) [22, 23].

Multi‐shot EPI techniques are vulnerable to phase errors resulting from bulk motion in between shots, leading to ghosting artifacts. These sequences therefore typically require the acquisition of navigator echoes to correctly register the shots in *k*‐space [24]. RS‐EPI is less vulnerable than interleaved EPI to gaps in k‐space left by the navigator correction process, as $k_{\mathrm{PE}}$ is fully sampled in every shot, and $k_{\mathrm{RO}}$ can be oversampled in each shot without significantly sacrificing readout time [23]. Conversely, the very short readout duration of each k‐space line in RS‐EPI requires more extensive ramp sampling, in which k‐space points are sampled while the readout gradient is ramping up or down. This reduces the effective readout gradient strength and means that $k_{\mathrm{RO}}$ is read out more slowly. RS‐EPI is thus less efficient than single‐shot or interleaved EPI at reducing $T_{2}^{*}$‐blurring. Nevertheless, the higher resolution and resilience against geometric distortion offered by RS‐EPI has made it a popular acquisition modality in body dMRI, and the technique has been validated in the breast [25], pelvis [26], and abdomen [27], where studies of qualitative imaging metrics, such as motion artifacts, geometric distortions, ghosting artifacts, and lesion conspicuity were rated more favorably in RS‐EPI than single‐shot EPI by experienced raters. These studies acknowledged the increased total scan time required for RS‐EPI [26, 27] as a limitation and potential barrier to wider use in clinical studies. In the liver, where respiratory motion is significant, single‐shot EPI resulted in fewer motion artifacts and more consistent ADC values than RS‐EPI [28, 29].

Reduced field‐of‐view (FOV) EPI (also known as ZOOM‐EPI) offers another method for reducing distortion in EPI. This can be achieved by applying 2D selective RF excitation pulses [30], suppressing the signal from outside the volume of interest [31], or both. These methods are useful in anatomical regions where there are large $B_{0}$ inhomogeneities, or where the target volume is small in one or more dimensions [20].

## 3 Non‐Standard Diffusion Imaging

### 3.1 Alternative Diffusion Sensitization Methods

The diffusion sensitizing gradients can be altered from the standard pair of gradient pulses used in the PGSE sequence, in order to achieve specific goals or overcome certain limitations of the standard method. Table 1 summarizes the methods described in this section.

**TABLE 1:** Summary of the alternative diffusion sensitization methods discussed in this review.

Method	Advantages	Disadvantages	Example applications
Bipolar diffusion gradients [32]	Reduced motion‐related distortion, up to second order	Increased TE Decreased *b*‐value	Heart [33] Abdomen [34]
Oscillating gradients [35]	Increased sensitivity to diffusion on smaller length scales Cell size estimation	Lack of sensitivity to higher Δ diffusion Increased TE Increased gradient duty cycle	Tumor cell size estimation (e.g., prostate cancer [36, 37], breast cancer [38])
Spherical tensor encoding [39]	Sensitivity to MD and anisotropy from a single acquisition	Increased TE	Heart [40] Abdomen [41] Prostate [42]

*Note:* The advantages and disadvantages are described relative to monopolar pulsed gradient diffusion encoding (Stejskal‐Tanner scheme). Example applications to body dMRI are non‐exhaustive.

#### 3.1.1 Bipolar Diffusion Gradients

The diffusion‐encoding gradients that sensitize the signal to microscopic diffusion of water molecules also sensitize the signal to bulk motion of tissue: if the sample moves en masse between the two gradient pulses (i.e., during time Δ), signal will be lost. Replacing the diffusion‐encoding gradients with a pair of bipolar gradient lobes on either side of the refocusing pulse (Figure 1B), provides flow compensation by nulling the first order moment of the gradient: the sequence is insensitive to motion while ∫Gtdt=0 [32]. Second‐order gradient moment nulling is also possible with the application of asymmetric bipolar pulses, for example where the paired positive gradient lobes have a different total area than the paired negative gradient lobes. Higher‐order motion compensation is possible with additional pulses, typically in a steady‐state or stimulated echo scheme, although this comes at the cost of increased effective TE or decreased *b*‐value [33].

This technique has found greatest use in the heart [32, 33, 43], but gradients can also be designed to suppress blood signals, enabling more accurate quantification of tissue diffusion parameters, especially in the liver [34].

#### 3.1.2 Oscillating Gradients and Time‐Dependent Diffusion

Replacing the pulsed gradients from the PGSE sequence with an oscillating waveform introduces sensitivity to diffusion on a shorter time scale than in the standard PGSE sequence [35]. This is illustrated in Figure 1C, which shows multiple short gradient pulses of alternative polarity before and after the refocusing pulse. The very short interval between two consecutive gradient pulses, Δ, means that the diffusion signal is sensitive to water molecules in highly restricted compartments, on the scale of ones of microns [44]. Applying this short pair of pulses multiple times increases the diffusion weighting of the signal, and enables reaching higher effective *b*‐values. The resulting oscillating‐gradient dMRI can be sensitized to microscopic structures of particular size by varying the oscillation frequency [44].

Acquiring both oscillating‐ and pulsed‐gradient dMRI data further enables the estimation of diffusion distances, which have been correlated with microstructural tissue properties such as cell size [45]. This technique—known as “time‐dependent diffusion spectroscopy”—has been applied in the body to estimate cancer cell size in prostate [36, 37] and breast [38] tumors.

#### 3.1.3 Spherical Tensor Encoding

Applying a symmetric pair of diffusion‐encoding gradients (as in the PGSE sequence) introduces a sensitivity to diffusion in a particular direction. This is not required to be along the principal coordinate axes, as a linear combination of physical gradients can achieve diffusion sensitization in any direction. With this approach, quantifying MD requires at least three separate acquisitions with different linear encoding directions [18].

Mori and van Zijl showed that applying bipolar gradients in all three directions subsequently achieves “trace‐weighting” without any contamination by off‐diagonal tensor elements [39]. For more time‐efficient spherical tensor encoding, gradients can be applied in multiple directions simultaneously, in patterns designed to cancel out contributions from off‐diagonal terms [46]. Nery et al. [41] used generalized gradient waveforms to efficiently provide spherical tensor encoding, and applied this technique to quantify microscopic FA in the kidneys. A similar approach has been used in cardiac [40] and prostate [42] dMRI.

### 3.2 Alternative Signal Generation Schemes

The 180° refocusing pulse followed by readout can be augmented or replaced, giving rise to several alternative schemes that have different advantages and applications in body dMRI. Table 2 summarizes the methods described in this section.

**TABLE 2:** Summary of the alternative signal generation schemes described in this review.

Method	Advantages	Disadvantages	Example applications
Twice‐refocused spin echo [47]	Reduced eddy current distortion Reduced motion‐related distortion	Increased TE Increased RF power deposition Creation of stimulated echoes	Heart [33]
Stimulated echo [48]	Reduced *T*_2_ dependence Intrinsic cardiac gating Reduced RF power deposition	Decreased SNR Increased bulk motion sensitivity Additional diffusion encoding by imaging gradients	Heart [33] Prostate [49, 50]
Turbo spin echo [51]	Higher resolution imaging Reduced susceptibility distortions	Increased RF power deposition Reduced SNR‐time efficiency Additional diffusion encoding by imaging gradients	Abdomen [52] Prostate [53]
Diffusion‐weighted SSFP [54]	Increased diffusion sensitivity Increased SNR‐time efficiency Enables 3D readout	Increased bulk motion sensitivity Increased off‐resonance effects Indirect quantification of diffusion parameters	Breast [55, 56] Extremities [57]
Diffusion‐prepared SSFP [58]	Enables motion‐compensated diffusion sensitization Enables balanced‐SSFP readouts	Increased bulk motion sensitivity Increased off‐resonance effects Increased signal dependence on *T*_1_ and *T*_2_	Heart [43] Abdomen [59] Prostate [60]

*Note:* The advantages and disadvantages are described relative to PGSE. Example applications to body dMRI are non‐exhaustive.

#### 3.2.1 Twice‐Refocused Spin Echo

The diffusion sensitizing gradients in a PGSE sequence are of the same polarity, and so both contribute to eddy current build‐up in the same way. This can lead to spatial distortions in the resulting image, especially if acquired with EPI. A solution to this is the twice‐refocused spin echo (TRSE) sequence, which features two 180° refocusing pulses and four diffusion gradients, in matched, opposite pairs, resulting in zero net eddy current [47]. The diffusion gradients can also be designed to achieve motion nulling (Section 3.1.1). This technique has been used in cardiac dMRI [33], including in combination with multi‐shot or steady‐state readouts (Section 3.2.5, below).

#### 3.2.2 Stimulated Echoes

The PGSE, strong diffusion encoding (high *b*‐value) requires increasing TE to allow for longer Δ (and δ), which results in greater *T*_2_ decay of the signal. The stimulated echo sequence (STE, also known as STEAM; schematically represented in Figure 1D) mitigates this by replacing the 180° pulse with a pair of 90° pulses, separated by a mixing time $t_{M}$. In this sequence, magnetization is effectively “stored” in the longitudinal direction during $t_{M}$, where it decays with *T*_1_ (typically ≫ *T*_2_). Once returned to the transverse plane, the signal refocuses to form a stimulated echo, which can be read out using a single‐shot or line‐reading imaging technique.

STE is most frequently used in cardiac dMRI, where it is typically combined with a bipolar diffusion‐sensitizing gradient, EPI readout, and DTI analysis [17]. Here the very short *T*_2_ of the myocardium is overcome, and cardiac motion can effectively be frozen, by beginning $t_{M}$ at a specific point in the cardiac cycle, and reading out the signal at the same point in the following cycle [48].

The lower energy deposition of STE, as opposed to SE, makes it well suited for multi‐shot acquisitions, such as the turbo‐STEAM sequence used by Merrem et al. [49] for prostate dMRI with reduced susceptibility artifacts. Similarly, Dan et al. [50] used a multi‐echo STE acquisition to quantify the TE‐dependence of ADC in the prostate. STE has also been used with oscillating diffusion gradients for time‐dependent dMRI [36], and with spiral readouts to further reduce TE, limiting *T*_2_ decay and improving SNR [61, 62].

The principal limitations of STE dMRI come from:

1. The intrinsically lower signal of a stimulated echo (half that of a spin echo) [12]. This is overcome in part by the significantly shorter TE that is possible, although the introduced *T*_1_‐weighting may lead to differing quantitative parameter estimates, as reported by Stabinska et al. [63] in their comparison of renal dMRI with PGSE and STE.
2. The increased sensitivity to motion over the longer $t_{M}$ [48]. In cardiac dMRI this means that while STE is robust to cardiac motion, it requires breath‐hold or navigator gating to ensure that respiratory motion during $t_{M}$ does not corrupt the signal [64]. It may be that for patients with arrhythmia or difficulties in performing a breath hold, that a spin echo‐based sequence, with motion‐compensated diffusion gradients (Section 3.1.1) is more suitable [65].
3. The greater effect of imaging gradients on the diffusion weighting, compared with SE [63]. Unbalanced gradients, such as for slice selection or signal spoiling, also contribute to the diffusion‐weighting of the signal, if applied during $t_{M}$, and their effect is greater with longer diffusion time [66]. This also results in differences in parameter values between STE and PGSE, and typically requires acquisition of additional diffusion directions for accurate calculation of DTI parameters.

#### 3.2.3 Turbo Spin Echo

Turbo spin echo (TSE; also known as FSE, RSE, and RARE) [51] is an extension to the standard PGSE sequence, in which the first refocusing‐pulse‐and‐readout pair is followed by a series of subsequent 180° RF pulses followed by readouts. Typically, a single *k*‐space line is read out per echo, which significantly shortens the readout duration, relative to EPI, and therefore reduces the effect of phase accumulation and distortions across the $k_{\mathrm{PE}}$ direction, while allowing for higher resolution imaging [53].

A consequence of adding diffusion‐sensitizing gradients to a TSE sequence is the violation of the Carr‐Purcell‐Meiboom‐Gill (CPMG) condition, which requires that all spins be fully refocused by each 180° RF pulse [51]. When this is not the case, additional stimulated echoes will be formed after each refocusing pulse, which may destructively interfere with subsequent spin echoes. The motion of spins under the diffusion‐sensitizing gradients violates this condition, necessitating the use of crusher gradients around subsequent refocusing RF pulses [52]. These additional gradients significantly lower the available signal in TSE, leading to lower SNR‐time efficiency, compared with EPI. This, along with the greater energy deposition necessitated by large numbers of 180° RF pulses, is a major limitation of diffusion‐prepared TSE.

In the body, TSE has been employed in prostate dMRI [67], including by Pirasteh and colleagues [53], who found that employing a radial *k*‐space sampling scheme (Section 3.3.1) improved image interpretability over EPI. In abdominal imaging, Gibbons et al. [52] found that ADC values were different with TSE as opposed to single‐shot EPI, possibly because of additional effective diffusion‐weighting caused by the additional gradients needed to uphold the CPMG condition.

#### 3.2.4 Steady‐State Free Precession

Steady‐state free precession (SSFP) imaging is defined by very short TR, on the order of $T_{2}$, and a series of α° RF pulses that tip a fraction of the magnetization into the transverse plane every iteration. This leads to a large number of coherence pathways of spin‐ and stimulated‐echoes that can be read out after each excitation (as shown in Figure 1E). Adding a single diffusion‐sensitizing gradient pulse introduces diffusion‐weighted contrast to the steady‐state magnetization, which accumulates through coherence pathways, leading to diffusion‐weighted (DW)‐SSFP [54, 68]. DW‐SSFP enjoys SNR gains due to the increased readout time, relative to Δ and TR, as well as greater sensitivity to diffusion than the PGSE preparation.

A principal disadvantage of DW‐SSFP is its sensitivity to motion within TR: A large number of repetitions is necessary to acquire a full 3D volume, and any motion between shots will cause phase inconsistencies, leading to image artifacts. These are typically dealt with retrospectively, through the use of phase navigators acquired for every shot [68]. Furthermore, banding or striping artifacts in SSFP images, caused by off‐resonance effects, may be more prevalent in body applications where there are large differences in susceptibility [69].

The quantitative use of DW‐SSFP to assess tissue parameters such as ADC is further complicated by the non‐linear relationship between gradient strength G and actual diffusion sensitization b, as well as by the sensitivity of the sequence to $T_{1}$, $T_{2}$, and α [70]. In applying DW‐SSFP to imaging the extremities, Chhabra and colleagues [57] actually found the latter to be advantageous, as the compounded $T_{2}$ and ADC contrast improved nerve identification, though other studies have recognized this as a confound of accurate diffusivity measurements [71].

#### 3.2.5 Diffusion‐Prepared Steady State

The DW‐SSFP technique described above (Section 3.2.4) applies a diffusion‐sensitizing gradient in every shot during the SSFP train, to achieve a high diffusion encoding in the steady state. An alternative technique is diffusion‐prepared SSFP (DP‐SSFP), in which a single diffusion preparation module (such as PGSE) is applied to the whole volume, prior to a 3D SSFP imaging train [58]. This enables a diffusion weighting based only on the preparation, and the ability to incorporate motion‐compensation and balanced SSFP readouts [68], while overcoming some of the disadvantages of EPI readout such as $T_{2}^{*}$‐blurring and geometric distortions.

Nguyen et al. [33, 43] used DP‐SSFP with a twice‐refocused preparation (Section 3.2.1) and second‐order motion compensation for DWI and DTI of the heart, with reduced motion artifacts compared with PGSE. DP‐SSFP techniques have also been applied to the prostate and abdomen, and were found to result in higher SNR, fewer distortions, and better visual quality than diffusion‐preparation with EPI readout [59, 60, 72].

### 3.3 Alternative Image Readout Techniques

Finally, it is possible to fill *k*‐space in ways that diverge from the widely used Cartesian approaches described above. Three broad families of trajectories have been used in the body (summarized in Table 3).

**TABLE 3:** Summary of the alternative image readout techniques described in this review.

Method	Advantages	Disadvantages	Example applications
Readout‐segmented EPI [22]	Increased resolution Reduced geometric distortions Reduced Nyquist ghosting	Increased sensitivity to bulk motion Increased $T_{2}^{*}$ blurring Increased total scan duration Increased eddy‐current induced distortions	Breast [25] Abdomen [28] Pelvis [26]
Radial [73]	Reduced TE Increased SNR Increased resolution Reduced impact of motion artifacts	Requires non‐Cartesian reconstruction Increased total scan duration Susceptibility to chemical shift artifacts	Prostate [49]
PROPELLER [74]	Increased resolution Reduced sensitivity to magnetic field inhomogeneity and eddy currents Reduced sensitivity to motion	Increased total scan duration Requires non‐Cartesian reconstruction	Abdomen [75] Pelvis [76]
Short‐axis PROPELLER [77]	Reduced geometric distortions Reduced Nyquist ghosting	Increased $T_{2}^{*}$ blurring Increased total scan duration Requires non‐Cartesian reconstruction	Heart [78]
Single‐shot spiral [79]	Reduced TE Efficient gradient usage Reduced eddy currents	Susceptible to blurring and distortions from gradient deviations Increased sensitivity to magnetic field inhomogeneities Requires non‐Cartesian reconstruction	Heart [61]
Interleaved spiral [80]	Reduced TE Self‐navigating Reduced readout duration Efficient gradient usage	Requires non‐Cartesian reconstruction Susceptible to blurring and distortions from gradient deviations Increased sensitivity to magnetic field inhomogeneities Increased total scan duration	Heart [62] Breast [55] Prostate [81]

*Note:* The advantages and disadvantages are described relative to single‐shot EPI. Example applications to body dMRI are non‐exhaustive.

#### 3.3.1 Radial

In radial acquisitions (also referred to as “stars” or “vanes”) data is read out along lines passing through the origin in *k*‐space, such that the sampling pattern resembles spokes on a wheel (Figure 2D). Since *k*‐space is filled in multiple lines, as opposed to a single shot, this technique tends to be used alongside line‐scanning based excitation schemes such as multi‐STE, TSE, or SSFP. As such, radial acquisitions gain the SNR benefits of short TE and a high acquisition duty cycle. Like all multi‐shot acquisitions, radial sampling is susceptible to motion between shots; however, unlike in Cartesian sampling, where motion results in ghosts in the phase‐encoding direction, motion in radial sampling results in tangential streaks which are generally less visually impactful [13].

The earliest application of radial dMRI in the body used projection reconstruction [73]. Radial (or other non‐Cartesian) data can also be reconstructed using more typical 2D or 3D FT methods. This can be done by phase conjugate reconstruction [82] or model‐based, regularized reconstruction [49]. Most commonly, non‐Cartesian data are first interpolated onto a Cartesian grid (“regridding”), at which point the conventional fast Fourier transform can be used [83].

For each line of radial data acquired, motion‐induced phase shifts can be corrected by filtered back‐projection, without leaving holes in *k*‐space which would blur the resulting image [13]. The information‐rich center of *k*‐space is also more densely sampled than the edges, and in the case of TSE or multi‐STE acquisitions, it is sampled at multiple effective TEs. Since image resolution depends on the extent of *k*‐space sampled, high resolution radial images can be acquired without incurring the $T_{2}^{*}$ blurring which affects single‐shot acquisitions with long readout times. Modern compressed‐sensing undersampling methods can be used to significantly decrease total scan time [49], and in prostate dMRI, multiple studies have exploited radial acquisition's robustness to susceptibility‐based distortions, compared with EPI [53, 84].

#### 3.3.2 PROPELLER

The periodically rotated overlapping parallel lines with enhanced reconstruction (PROPELLER, also referred to as “BLADE”) method is to radial sampling as segmented EPI is to Cartesian line‐based sampling. As originally described by Pipe in 1999, PROPELLER involves collecting a “blade” of lines in *k*‐space using an fast readout (such as TSE or EPI), and then in the following shot, rotating the blade about the center of *k*‐space and acquiring again (Figure 2E) [74]. This process results in a central circle which has been repeatedly sampled, and more sparse sampling of the higher frequency areas. Phase errors due to eddy currents or inter‐shot motion can be corrected because the central circle acts as a navigator. PROPELLER offers higher resolution and lower sensitivity to field inhomogeneity than EPI, as well as robustness to eddy current warping and bulk motion [85].

In the body, PROPELLER was first used for DWI in the abdomen by Deng and colleagues [86], who found that a TSE‐based PROPELLER sequence resulted in less blurring, better anatomical detail, and higher lesion conspicuity in hepatocellular carcinoma than single‐shot EPI with the same read time. Similar improvement in image quality was found with PROPELLER sequences in pelvic imaging [87, 88].

In the “short‐axis” variant of PROPELLER, each blade is acquired using an EPI trajectory which traverses a short *k*‐space distance in the readout direction, with a larger number of phase blips to cover the entirety of $k_{\mathrm{PE}}$ [77]. This method is thus analogous to readout‐segmented EPI. The increased effective bandwidth in the $k_{\mathrm{PE}}$ direction reduces geometric distortions without extending the total readout time. Sadighi and colleagues [78] combined this readout with second‐order motion‐compensated diffusion‐sensitizing gradients to produce 3D cardiac DTI data that were robust to motion and exhibited far fewer aliasing artifacts than single‐shot EPI.

PROPELLER is not as time‐efficient as some other sampling methods, given the high degree of oversampling in the central circle [86], and suffers from lower SNR in the edges of *k*‐space, compared to Cartesian methods [85]. Furthermore, the quantitative ADC values obtained using PROPELLER were found to be significantly different from those obtained by EPI‐based methods, as reported in the liver [86] and rectal tumors [30].

#### 3.3.3 Spiral

Typically, the most important information in an image is encoded at the low‐frequency center of *k*‐space. Relaxation processes mean that increasing TE (the time between excitation and when the center of *k*‐space is read out) decreases available signal, and hence decreases SNR. Higher TE also leaves more time for bulk motion, which degrades image quality. In dMRI, as in most other applications, a TE that is as short as possible is usually preferred. This is where a spiral trajectory (Figure 2F) [79] offers a significant advantage over Cartesian readouts, since in spiral, the information at the center of *k*‐space is acquired at the beginning of the readout, rather than in the middle [80]. Unlike radial readouts, spiral trajectories are able to cover all of k‐space in a single shot, removing the need for shot‐to‐shot navigators and enabling slice‐by‐slice imaging. Spiral trajectories also utilize gradients more efficiently, compared to the sharp changes of direction required in Cartesian trajectories, and cover *k*‐space in a more effective elliptical shape [80, 82].

As with EPI, single‐shot spiral acquisitions suffer from long readout times, and are therefore vulnerable to $T_{2}^{*}$‐related signal loss. Eddy currents, gradient system imperfections (including the use of waveforms that typical scanner gradient systems are not optimized for) and concomitant fields can lead to trajectory errors, which can cause severe distortions in the resulting images [82]. Off‐resonance effects due to $B_{0}$ inhomogeneities and susceptibility differences can be more severe in spiral images compared to Cartesian, because effects are blurred across both in‐plane spatial directions, rather than only the phase‐encoding direction. Phase accumulation due to concomitant fields also produces a more severe distortion than is typically seen with EPI [82].

The trade‐off between readout duration, resolution, and $T_{2}^{*}$‐blurring can be improved through the use of interleaved spiral acquisitions [80, 89]. Analogous to segmented EPI (Section 2.3) these are susceptible to bulk motion and require either navigators or additional registration steps. However, since the center of *k*‐space is sampled with every shot, self‐navigation is possible, especially if variable‐density trajectories are used to acquire sufficient data from the center of *k*‐space each time [62, 80].

The challenge of correcting spiral trajectories can be overcome through trajectory measurements using a phantom, or through acquiring additional field mapping images in vivo prior to data acquisition. Frequency‐segmented reconstruction can be used to overcome off‐resonance effects and the fact that non‐Cartesian data cannot be fast Fourier transformed [90]. Discrete Fourier transforms based on phase conjugate reconstruction, or regularized model‐based reconstructions are also possible, but interpolation followed by fast Fourier transform is the preferred approach [55, 82].

A small number of recent works have employed spiral trajectories in body dMRI, with variations in diffusion preparation and image encoding. Gorodezky and colleagues [61] used a single‐shot spiral readout following an STE sequence for cardiac DTI. They observed a marked SNR increase with spiral over EPI, which exceeded the theoretically expected SNR gain from reduced TE alone, perhaps due to other differences in acquisition and reconstruction. They also investigated interleaved, variable‐density spirals in a self‐navigated multi‐shot paradigm and achieved higher imaging resolution without compromising image quality [62].

Moran et al. [55] used a DW‐SSFP approach, acquiring a single 3D spiral readout per TR, with diffusion sensitization gradients in every repetition for a high *b*‐value equivalent. This 3D “cones” approach was self‐navigated, allowing for greater robustness to motion than EPI, and the steady‐state acquisition resulted in high lesion conspicuity.

Recently, Molendowska et al. [81] demonstrated high resolution, high quality SE spiral dMRI images in the prostate. These authors used external field probes to correct for off‐resonance effects and trajectory errors [91], which are not yet widely available, and a specialist Connectome scanner with high‐performance gradients.

## 4 Applications in the Body

This section reviews the application of non‐standard dMRI techniques in the body and is organized by the anatomical region of interest.

### 4.1 Abdomen

DMRI has the potential to shed additional light on diseases of the abdominal organs without the need for exogenous contrast agents and has been widely integrated into routine clinical protocols [2, 5, 6]. There are particular challenges presented by this anatomical region.

The large size of the liver necessitates a large FOV (hence, typically, a large number of phase encoding lines), which means more potential for susceptibility‐related distortions in EPI [92]. The large blood volume of the liver also contributes to the diffusion‐weighted signal. This is typically nulled using motion‐compensated diffusion gradients (Section 3.1.1) [34, 93]. The proximity to the lungs means that abdominal imaging is also affected by respiratory motion. Lee et al. [59] combined bipolar motion‐compensated gradients with a DP‐SSFP paradigm (Section 3.2.5) and irregular Cartesian readout for high resolution whole‐abdomen coronal imaging. Their readout filled *k*‐space non‐uniformly across several shots, with central samples acquired at the beginning of each steady‐state readout train. This allowed for self‐navigation between shots, leading to free‐breathing acquisition. Similarly, Liang et al. [75] acquired high quality free‐breathing liver images using PROPELLER (Section 3.3.2) to navigate between shots.

Quantifying disease‐related changes in tissue microstructure can be confounded by normal physiological processes in the abdominal organs [6], making more advanced diffusion models (such as IVIM, DTI, and DKI) especially useful [5]. These techniques, however, require several diffusion‐weighted volumes with different *b*‐values and/or orientations, making motion correction and registration crucial. Nery et al. [41] used linear and spherical diffusion tensor encoding (Section 3.1.3) to measure microstructural FA, with minimal *b*‐value and acquisition time, more reliably and informatively than with linear encoding alone. In terms of the signal generation scheme, Stabinska and colleagues [63] found that PGSE outperformed an STE acquisition (Section 3.2.2) in renal DTI, in terms of SNR and anatomical delineation in the resulting ADC maps.

### 4.2 Pelvis

The pelvic region contains a number of organs and glands of significant clinical importance, for which dMRI can offer valuable information alongside other imaging modalities [14]. The pelvis presents challenges to imaging, such as large susceptibility differences between tissue, air (rectal gas), bone, and often surgical material [15]. These cause distortions in EPI [14], and also lead to blurring and other artifacts in non‐Cartesian acquisitions such as spiral [81]. Some susceptibility artifacts can be mitigated by patient preparation: administration of spasmolytics and an enema prior to the scan, to remove rectal gas and reduce motion associated with bowel function [3], although this may be considered excessively invasive in screening scans and adds potential contraindicators which may exclude some patients from MRI altogether. The anatomical position of tissues of interest (particularly the prostate) far away from the surface of the body limits SNR when using traditional external coils. Using an endorectal coil improves signal acquisition, but precludes the use of parallel imaging techniques that rely on multiple coils [14].

Several non‐standard dMRI techniques have been employed to meet these challenges. Spin‐warp image encoding schemes are much less affected by susceptibility artifacts and motion than single‐shot techniques. DP‐SSFP sequences (Section 3.2.5) have been used in combination with 3D Cartesian [60] and 3D SECTOR [72] readouts in prostate imaging. SECTOR, which samples the center of *k*‐space in every shot, helped to reduce motion and eddy‐current induced errors, and helped to mitigate *T*_1_ confounds by acquiring the center of *k*‐space at the beginning of the readout. DP‐SSFP resulted in higher resolution than EPI, fewer visible artifacts, and greater sensitivity and specificity in identifying lesions [60]. TSE sequences (Section 3.2.3) have also been used with 3D Cartesian and 2D radial readouts [53]. Crop and colleagues [94] used SPLICE with DW‐TSE in prostate cancer patients, overcoming violation of the CPMG condition, albeit at a minor cost of time or SNR efficiency. Lee et al. [87] compared TSE with PROPELLER readout against readout‐segmented EPI, in evaluating pelvic bone metastases, which are often small in size and obscured by geometric distortions. They found that TSE‐PROPELLER was less affected by motion, and had increased SNR with better lesion visibility, although it required a longer scan time than EPI. Similarly, Czarniecki and colleagues [76] used PROPELLER (Section 3.3.2) to acquire prostate images with fewer distortions than EPI, in patients with metal pelvic implants.

**FIGURE 3:**
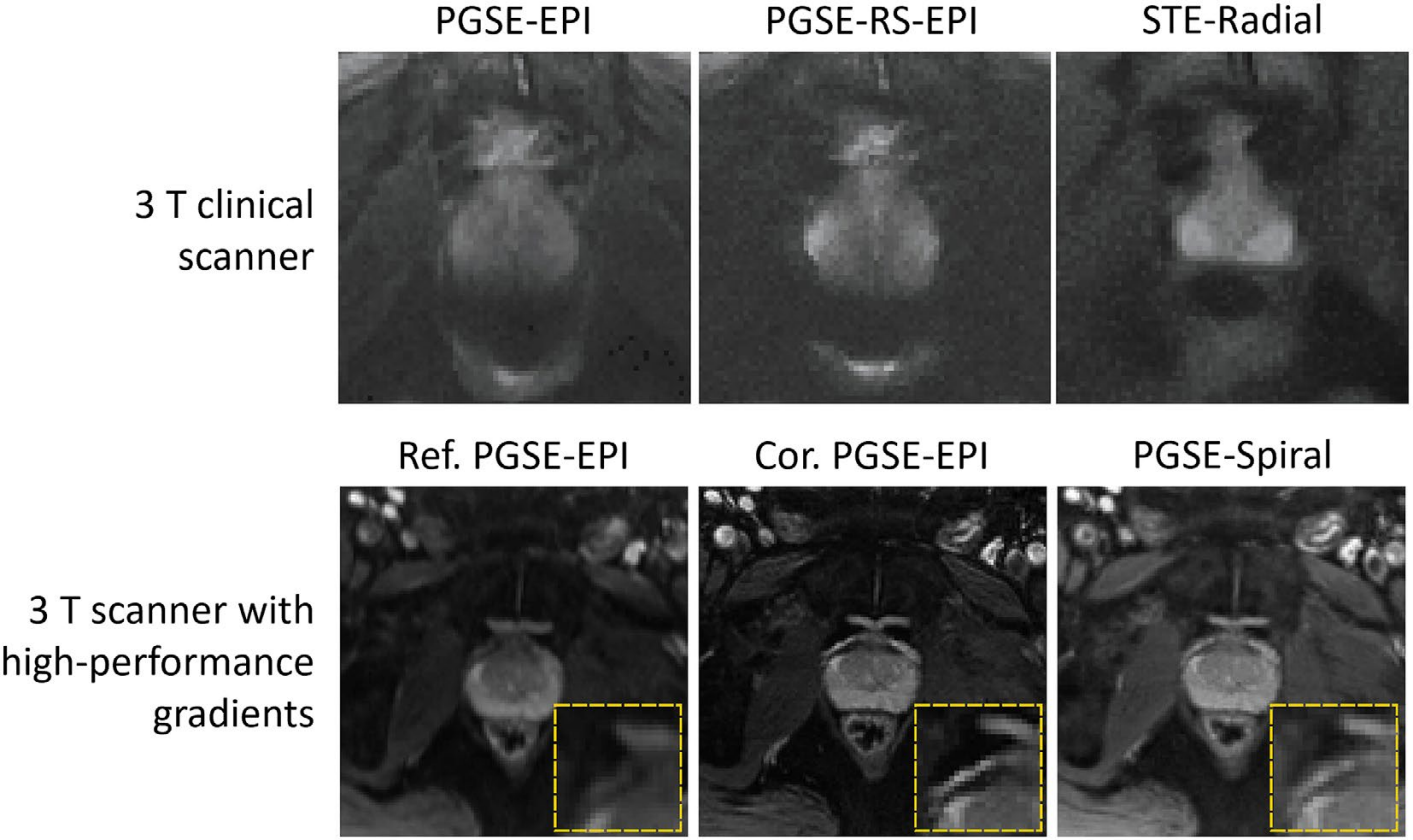
Example diffusion‐weighted images of the prostate, comparing image acquisition methods. Top row (left–right): PGSE‐EPI, PGSE‐RS‐EPI, and STE with radial readout. Images adapted from fig. 4 in Merrem et al. [49] (https://doi.org/10.1002/nbm.4074), reproduced with modifications with permission from John Wiley and Sons. All rights reserved. Bottom row (left–right): Vendor‐provided (reference) PGSE‐EPI; field‐map corrected (cor.) PGSE‐EPI; PGSE with spiral readout. Images adapted from fig. 5 in Molendowska et al. [81] (https://doi.org/10.1002/mrm.30351), licensed under CC BY 4.0 (https://creativecommons.org/licenses/by/4.0/); modifications were made.

A single‐shot spiral readout, tested by Molendowska et al. [81] on a high‐performance gradient system, enabled high resolution prostate dMRI with fewer motion artifacts and higher SNR than EPI, though this also required the use of external field probes for higher‐order corrections. Reduced FOV techniques have also been applied in the prostate to attain higher resolution with fewer susceptibility artifacts [88]. Example prostate dMRI images with different readout techniques are shown in Figure 3.

In quantitative prostate dMRI, Wu et al. [37] used time‐dependent dMRI with oscillating gradients (Section 3.1.2) to probe intracellular volume fraction and cellularity, which were both found to correlate with histological grading of prostate tumors.

Beyond the prostate, Matsumoto et al. [30] compared PROPELLER and conventional single‐shot EPI readouts and reduced FOV EPI with spatially‐selective RF pulses for rectal dMRI. They found that reduced FOV was able to achieve greatest image sharpness, but that the PROPELLER readout reduced motion artifacts and susceptibility distortions significantly, although they note that the ADC values obtained from the three methods were not equal, highlighting the importance of validation of quantitative values.

### 4.3 Breast

Breast cancer is the most common cancer among women, and remains the fifth leading cause of cancer‐related deaths worldwide [95], despite the improved survivability afforded by widespread screening using mammography. DMRI results in better lesion detection than mammography or ultrasound, and the strong correlations between ADC and microstructure mean that dMRI also offers the potential for tumor characterization and prognosis [16]. Single‐shot PGSE‐EPI is the most common technique for breast dMRI, and consensus recommendations center around EPI with conventional or segmented readout [11]. However, as in other anatomical regions, EPI suffers from geometric distortions due to susceptibility differences, and typically requires a trade‐off between short readout time (hence, robustness to motion) and low resolution [55].

Steady‐state acquisitions (Section 3.2.4) have been used to increase resolution and reduce distortions. Granlund et al. [56] report a modest increase in scan time per average with 3D DW‐SSFP compared to PGSE‐EPI, but an increase in effective SNR per unit time, and images that were preferred by radiologists. Similarly, Moran et al. [55] used a multi‐shot 3D spiral (“cones”) readout (Section 3.3.3) with DW‐SSFP, and found that this method improved image quality and reduced artifacts due to motion and susceptibility. Zhou et al. [96] improved the motion robustness of breast dMRI using EPTI, which also reduced susceptibility‐induced distortions, despite the $B_{0}$ inhomogeneity surrounding the breast. To provide additional information about tumor environments, oscillating‐gradient dMRI (Section 3.1.2) has been used, in combination with SE and STE signal generation, to quantify cell size in breast tumors [38, 66].

### 4.4 Heart

**FIGURE 4:**
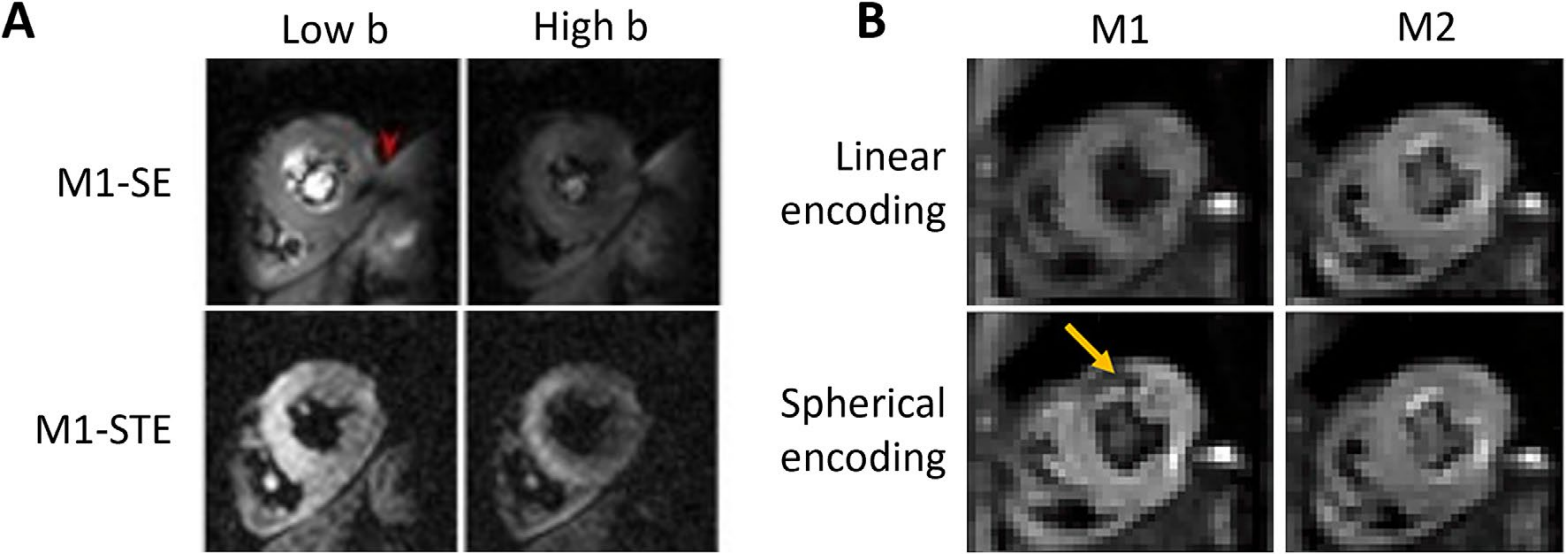
Example diffusion‐weighted images of the heart, comparing diffusion preparation methods. (A) First‐order motion nulled SE versus STE, for low and high *b* values. Images adapted from fig. 1 in Scott et al. [97] (https://doi.org/10.1016/j.jocmr.2024.101052). (B) Linear versus spherical encoding, for first‐ (M1) and second‐order (M2) motion‐nulling. Images adapted from fig. 4 in Lasič et al. [40] (https://doi.org/10.1002/nbm.4213). All images licensed under CC BY 4.0 (https://creativecommons.org/licenses/by/4.0/); modifications were made.

DMRI can be used to characterize the microstructure of the heart, especially given the anisotropic nature of the myocardium. Motion represents the largest challenge to cardiac imaging, but off‐resonance effects and tissue strain or deformation also impact the measured signal. Three main approaches have been used to mitigate the effect of cardiac motion in dMRI:

1. STE acquisition (Section 3.2.2) with cardiac gating. Here, ECG triggering is used to ensure that the two lobes of G are applied at the same phase of two consecutive cardiac cycles [48]. This approach allows for an extended mixing time $t_{M}$ leading to high *b*‐values, while keeping δ as short as possible to limit motion effects. The absence of 180° RF pulses also typically results in sharper slice profiles. However, this approach is limited by its lower SNR relative to SE, and its sensitivity to respiratory motion and tissue strain. Figure 4A shows example cardiac dMRI images with SE and STE [97].
  Additionally, the readout step is also affected by cardiac motion. To overcome the relatively long TE and readout duration of EPI, Gorodezky and colleagues [61, 62] employed a spiral readout trajectory (Section 3.3.3) for cardiac DTI. This achieved a shorter TE and super‐linear increase in SNR, relative to EPI with the same readout duration; however, actual values of DTI parameters were not consistent between methods [61]. Multi‐shot spiral acquisition (with field mapping for motion‐induced phase correction) enabled an increase in resolution by extending the readout of each spiral interleaf in k‐space [62].
2. Motion‐compensated diffusion encoding (Section 3.1.1). Dou et al. [32] employed a flow‐compensated bipolar encoding gradient, within an SE scheme, resulting in an SNR increase relative to STE. In contrast to these findings, Scott and colleagues [65, 97] showed that STE produced more consistent DTI parameter estimates throughout the cardiac cycle than motion‐compensated SE. Motion compensation up to second order has also been combined with spherical gradient tensors (Section 3.1.3) to produce maps of isotropic diffusivity in the heart, using a single‐shot EPI readout, examples of which are shown in Figure 4B [40].
3. Diffusion‐prepared SSFP (Section 3.2.5). This utilizes a much shorter echo train length than EPI, reducing the severe magnetic susceptibility induced distortion caused by air‐tissue interfaces in the lungs [33, 43]. Since this approach acquires each *k*‐space line within a single cardiac cycle, it is compatible with free breathing and heart rate pathologies such as arrhythmia.

## 5 Discussion

### 5.1 Advantages of Non‐Standard dMRI Acquisitions

Diffusion MRI offers a non‐invasive, non‐contrast method for probing tissue microstructural properties. Under its standard PGSE‐EPI acquisition, dMRI is widely used in clinical imaging protocols throughout the body, in a range of applications and pathologies. A myriad of variations on the PGSE‐EPI scheme are available, and many of them have been utilized in body imaging. These offer significant potential benefits and some trade‐offs.

Some methods directly address the traditional compromise between acquisition time and resolution—the improvement of one often being tied to the detriment of the other. Spin‐warp Cartesian readouts offer higher resolution than EPI, albeit at a cost of total acquisition time, as they require multiple excitations rather than a single shot [52]. Spiral readouts allow greater control over this balance, without compromising SNR: the center of *k*‐space is sampled first, granting a shorter TE (hence, increased SNR), while the tail of the spiral can be extended to any radius in *k*‐space, increasing resolution while increasing readout time [81]. Radial trajectories can achieve greater time efficiency, without compromising resolution, through the use of under‐sampling [49].

Other non‐standard methods aim to improve imaging quality by reducing distortions, which predominantly arise from three sources: (1) bulk tissue motion, (2) magnetic susceptibility differences, and (3) eddy currents.

1. Bulk motion can be compensated for through the use of bipolar diffusion gradients, which can null moments up to second order, offering a particular benefit in cardiac dMRI [43]. Spin‐warp readouts are more robust to short time‐scale motion than EPI, and SSFP in particular offers resilience to motion between diffusion encoding and readout [33]. Radial, PROPELLER, and spiral readouts sample the center of *k*‐space in every acquisition, and can therefore be self‐navigated and retrospectively motion‐corrected [74, 80, 98]. These methods have been used in the chest and abdomen to account for cardiac and respiratory motion. While previous work is limited, they could also be of use in pelvic imaging, in applications where pre‐scan spasmolytics cannot be used [3].
2. Magnetic susceptibility differences are a major issue in anatomical regions that contain multiple tissue interfaces, especially air‐tissue boundaries, or high fat content. The effect of susceptibility on phase accumulates over the readout time, and so EPI‐based sequences are more severely affected by these artifacts. Readout‐segmented EPI and spin‐warp readouts produce less distorted images [23, 53, 99]. Radial and multi‐shot spiral readouts are also robust against susceptibility‐related artifacts, and have been used in prostate with success [81, 84].
3. Eddy currents arise in the coils of an MR system whenever magnetic fields change, and these currents in turn generate magnetic fields which result in phase differences across the image. The switching of diffusion encoding gradients, in particular, generates eddy currents that produce distortions in subsequent EPI images. These can be canceled through the use of asymmetric bipolar diffusion gradients in a twice‐refocused spin‐echo sequence [33, 47]. Such sequences have been applied in cardiac and breast dMRI in combination with a steady‐state Cartesian readout [43, 56], or 3D spiral trajectory [55].

Finally, there are some non‐standard dMRI methods that offer different or additional information than would be obtained by PGSE alone: Oscillating diffusion gradients provide sensitivity to diffusion on much shorter distances than pulsed gradients, leading to cell size quantification which is particularly useful in oncology applications throughout the body [45]. When combined with gradient echo sampling, further relaxometry information can be incorporated [36, 100].

These concerns notwithstanding, the choice of dMRI acquisition method depends strongly on the target anatomy and its properties. As the organ with the most motion, the heart is typically imaged using readouts with short acquisition times that can be gated to the cardiac cycle: SSFP [33, 43] and multi‐shot spiral [61, 62]. Abdominal imaging is most affected by respiratory motion, which is slow enough to allow for single‐shot EPI and involves significant motion between excitation and readout. Bipolar diffusion gradients and double‐spin‐echo refocusing are often used here [34, 59]. Radial and PROPELLER readouts were most employed in pelvic imaging [30, 49, 53, 59, 76, 88], where large susceptibility differences between tissues would cause significant artifacts in EPI, but can be mitigated using these alternative trajectories.

### 5.2 Impediments to Wider Use

Despite these advantages, non‐standard dMRI acquisitions remain underutilized in most body applications. There are several possible reasons for this:

1. Total scan duration. Single‐shot EPI has the advantage of acquiring entire 2D slices per TR (typically on the order of 2–5 s [10]), enabling acquisition of 3D volumes with multiple *b*‐values and/or directions in a matter of minutes. Multi‐shot techniques such as RS‐EPI, PROPELLER, and interleaved spiral require multiple repetitions per slice. Spin‐warp signal generation schemes such as TSE and SSFP, which acquire a single line of *k*‐space per echo, can utilize a shorter TR per acquisition and can make use of 3D acquisition and reconstruction, but typically require a longer total scan duration than single‐shot EPI [43, 72].
2. Motion sensitivity. PGSE‐EPI has a short readout time per slice, which makes it relatively insensitive to bulk motion of tissue. Non‐standard acquisitions that involve reading out data across multiple shots are vulnerable to motion between shots, resulting in phase inconsistencies which corrupt the reconstructed magnitude images [68]. Multi‐shot acquisitions typically include *k*‐space navigators to correct the phase (and acquisitions such as PROPELLER and interleaved spirals are self‐navigated due to the fact that the center of *k*‐space is sampled in every shot), but these can still be corrupted by extreme motion [20]. STE encodes diffusion across pairs of adjacent repetitions, which means it is affected by motion between TRs. In cardiac dMRI, this makes breath‐holding necessary, which can limit the technique's applicability to clinical populations [64]. Driven equilibrium preparations are also affected, as the longitudinal magnetization can be corrupted by motion [9].
3. Image artifacts. Imaging techniques that require large readout gradient amplitudes, such as PROPELLER, are susceptible to Nyquist ghosting [77]. PROPELLER and interleaved EPI suffer from geometric distortions due to slow traversal of $k_{\mathrm{PE}}$. On the other hand, techniques which traverse $k_{\mathrm{PE}}$ quickly, such as RS‐EPI, rely on rapid gradient switching to do so, which leads to eddy current‐induced distortions [23]. Off‐resonance effects, caused by magnetic susceptibility differences or chemical shift, can result in more severe artifacts than in EPI: in SSFP, they lead to banding or striping artifacts rather than localized drop‐out [69], and in spiral, the effect can be distributed across the whole image [82]. Trajectory errors in spiral imaging can also cause severe blurring artifacts and distortion.
4. Differences in quantitative values. In applications where diffusion‐weighted images alone are not sufficient, accuracy and repeatability of quantitative parameters such as ADC, FA, or more advanced parameters are required. This is ruled out a priori in methods such as diffusion‐weighted SSFP, where the measured signal is a sum of contributions from different coherence pathways, which makes defining a single *b*‐value impossible [70, 71]. In methods such as STE, the differences in timing and the inclusion of $T_{1}$‐weighting confound quantitative measurements. Scott et al. [65] found this to be the case in cardiac DTI, with significant differences in MD and FA between STE and SE sequences. Similarly, in abdominal dMRI, Gibbons et al. [52] observed significant differences in ADC between TSE and SE‐EPI sequences, which were attributed to the non‐trivial effect of additional crusher gradients on the diffusion sensitization. Furthermore, alternative readout techniques have been found to result in ADC differences with no direct explanation, such as the differences between PROPELLER and EPI values that were observed in studies of the abdomen and rectum [30, 86].
5. Hardware and software availability. Hardware availability can dictate the range of techniques that centers can employ. Non‐standard readout trajectories such as spiral require higher gradient performance to achieve comparable resolutions to Cartesian encoding [81]. Oscillating gradient preparations also require high gradient slew rates [35]. In addition, many of the non‐standard acquisitions described above are not available as commercial products on most scanners, although improvements to the PGSE‐EPI acquisition such as (readout‐)segmented EPI and reduced FOV dMRI are now widely available [30, 88]. Furthermore, non‐Cartesian readouts require complex reconstruction pipelines involving either regridding, non‐uniform Fourier transforms [83], or model‐based approaches [82], which may not be available in‐line.

There have also been cases in which PGSE‐EPI has performed better than alternative methods in image quality and consistency. Stabinska and colleagues [63] found that PGSE produced ADC maps with better contrast between kidney regions, higher repeatability of ADC values between repeated acquisitions in the same subject, and higher subjective image quality on ADC and FA maps than STE, according to blinded ratings by experienced radiologists.

Recent efforts have been made to standardize dMRI acquisition in certain applications, such as the EUSOBI International Breast DWI working group's consensus recommendations published in 2020 [11]. The recommendations assume a PGSE preparation, while acknowledging the potential improvement offered by RS‐EPI, and prefer single‐shot EPI. They recognize that the lack of standardization across the literature makes it difficult to offer specific recommendations on the implementation of other sequences. Similarly, the 2020 consensus recommendations for renal DWI also endorsed single‐shot EPI with PGSE preparation as the preferred technique [10].

With that in mind, a recent review by Stabinska et al. [6] has highlighted the potential benefits of non‐standard acquisition techniques, especially PROPELLER, for advanced renal dMRI applications, with the acknowledgement that much more work is required for standardization.

## 6 Conclusion

This article has reviewed the ways in which non‐standard acquisition techniques (defined as those not using PGSE preparation and/or EPI readout) have been applied to non‐brain diffusion imaging. It is necessarily limited in scope, and for the sake of brevity and coherence some instances of non‐standard techniques have been excluded. These have included techniques that have not been employed recently, and applications that have fallen outside the major anatomical regions identified in Section 4. The use of these techniques in preclinical or pediatric and fetal imaging has also been excluded. A systematic evaluation of all of the strengths and weaknesses of the methods against one another is beyond the scope of this review.

It is hoped that this article has highlighted the potential for further development and deployment of advanced dMRI acquisition techniques throughout the body, and that next steps will include systematic evaluation and harmonization of methods that are appropriate to the most pressing applications of clinical imaging today.

## Funding

This work was supported by the European Research Council (101164674).

## Conflicts of Interest

The authors declare no conflicts of interest.
